# Mortality in Patients With Bipolar Disorder : An Experience From A Tunisian Psychiatric Hospital

**DOI:** 10.1192/j.eurpsy.2026.11282

**Published:** 2026-07-31

**Authors:** G. ltaief, A. Karmous, A. Touiti, N. Rajhi, M. Bellali, N. Bram

**Affiliations:** Forensic Psychiatry Department, Razi Hospital, Manouba, Tunisia

## Abstract

**Introduction:**

Studies on mortality among bipolar patients remain fairly limited, although several studies have shown that patients with bipolar disorder have a significantly higher mortality rate than the general population, with increased risks from both natural and unnatural causes.

**Objectives:**

The aim of our work was to study the mortality among patients with bipolar disorder in the psychiatric wards of Razi Hospital (Manouba, Tunisia).

**Methods:**

A retrospective descriptive study was conducted over an 11-year period, from January 2012 to December 2022, focusing on patients with bipolar disorder who died during their stay at Razi Hospital. Data were collected from patients’ medical records. Sociodemographic and clinical characteristics as well as the medico-legal causes of their deaths were gathered.

**Results:**

A total of 22 patients were included. The mean age was 49.32 ± 12.1 years. Seventeen patients (77.27 %) were male and five (22.73%) were female. Analysis of socio-economic status showed that 22.73 % of patients (N=5) came from a low socio-economic background. A history of a psychiatric illness in first-degree relatives was found in 36.36 % of cases (N=8) and in 31.82% (N=7) of cases in second-degree relatives. Nine patients (40.9 %) had a personal history of somatic illness. Two patients (9.09 %) had a criminal record. The cause of death was identified in seven cases (31.8 %), with six cases of natural death and one case of suicide by hanging. The autopsy results were available in 4 cases (8.1 %) including pulmonary embolism in one case, acute pulmonary oedema in one case and infectious pneumopathy in two cases.

**Conclusions:**

Our findings confirm that patients with bipolar disorder are at an elevated risk of premature mortality. This highlights the need for systematic monitoring, early detection of comorbidities, and the implementation of targeted prevention strategies within psychiatric settings.

**Disclosure of Interest:**

None Declared

